# Treemble: a graphical tool to generate Newick strings from phylogenetic tree images

**DOI:** 10.1093/bioinformatics/btag197

**Published:** 2026-04-22

**Authors:** John B Allard, Sudhir Kumar

**Affiliations:** Institute for Genomics and Evolutionary Medicine, Temple University, Philadelphia, PA 19122, United States; Department of Biology, Temple University, Philadelphia, PA 19122, United States; Institute for Genomics and Evolutionary Medicine, Temple University, Philadelphia, PA 19122, United States; Department of Biology, Temple University, Philadelphia, PA 19122, United States

## Abstract

**Summary:**

Phylogenetic trees are ubiquitous and central to biology, but most published trees are available only as visual diagrams and not in the machine-readable Newick format. There are, thus, thousands of published trees in the scientific literature that are unavailable for follow-up analyses, comparisons, and supertree construction. Experts can easily read such diagrams, but the manual construction of a Newick string from a diagram is laborious, error-prone, and time-consuming. Previous attempts to semi-automate the reading of tree images relied on image processing techniques. These often encounter difficulties as typical published tree diagrams contain various graphical elements and annotations that overlap the branches, such as error bars on internal nodes. Here we introduce Treemble, a user-friendly desktop application for generating Newick strings from tree images. The user simply clicks to mark node locations, assisted by a deep learning-based node detection tool, and Treemble algorithmically assembles the tree from the node coordinates alone. Treemble also facilitates the automatic reading of tip name labels and can be used for both rectangular and circular trees.

**Availability and implementation:**

Treemble is a native desktop application for macOS and Windows and is freely available, with documentation, at treemble.org. Source code is available at github.com/John-Allard/Treemble. The trained node detection model is available at huggingface.co/John-Allard/treemble-1.

## 1 Introduction

Evolution is the single most important unifying principle in the life sciences, and as such understanding it is key to all biological research, either directly or indirectly (Dobzhansky 1973). A crucial factor in understanding evolution is the ability to discern the ancestry and relationships of organisms and genes. Tree diagrams (phylograms or cladograms) are the most common way to present inferred evolutionary relationships, whether derived from traditional morphological character-based analyses or molecular phylogenetics (Nei and Kumar 2000). Vast numbers of such trees are published each year (the phrase “phylogenetic tree” produces over 2 million hits in Google Scholar). Phylogenetic tree figures typically communicate not only the inferred ancestral branching patterns (the tree topology), but also the branch lengths in units of the number of molecular substitutions per site, or may be time-calibrated and expressed in millions of years (Nei and Kumar 2000).

These phylogenies are typically generated using phylogenetic analysis software, which can export them in machine-readable Newick format (Felsenstein 1989) that encodes the topology and branch lengths. However, text files containing Newick tree(s) are frequently missing from the supplementary information of publications that display phylogenetic tree diagrams, as has been observed previously (Kumar et al. 2022). Therefore, we and other researchers wishing to use published phylogenies in downstream analyses need to translate graphical phylogeny displays into textual Newick representations. However, generating a Newick representation manually remains an arduous task, which we have found to require hours for trees with even a small number of tips (∼50) and much longer for big phylogenies.

For this reason, multiple software packages have been published to partially automate the process of acquiring a Newick representation from a tree image (Laubach and von Haeseler 2007, Hughes 2011, Laubach et al. 2012). Both TreeSnatcher (Laubach and von Haeseler 2007, Laubach et al. 2012) and TreeRipper (Hughes 2011) detect tree branches in a figure by image processing techniques to identify the foreground in the form of contiguous dark pixels against a light background. This can be effective for optimally clean and annotation-free phylogenies, but the success rate for TreeRipper was low due to the complexity of many images, and this web application is no longer available at the published URL (Hughes 2011). TreeSnatcher required the user to use image processing tools to condition the tree pixels to be detected, and in cases where extraneous intersecting lines, organismal silhouettes, and boxes are present, the user needed to manually delineate the foreground to remove such distractions and make other manual modifications to aid in detection of nodes. In addition, entry of tip names required manual typing.

Here, we present a new software package, Treemble, for building Newick text strings from phylogenetic tree images semi-automatically and quickly. Treemble does not rely on image processing, so tree displays adorned with a multitude of annotations can be processed quickly. Subtrees can also be captured. Treemble can handle images containing rectangular and circular phylogenies and measure branch lengths in the units of substitutions per site and time. In the following sections, we describe Treemble’s architecture, capabilities, and performance.

## 2 Results

**Figure 1:**
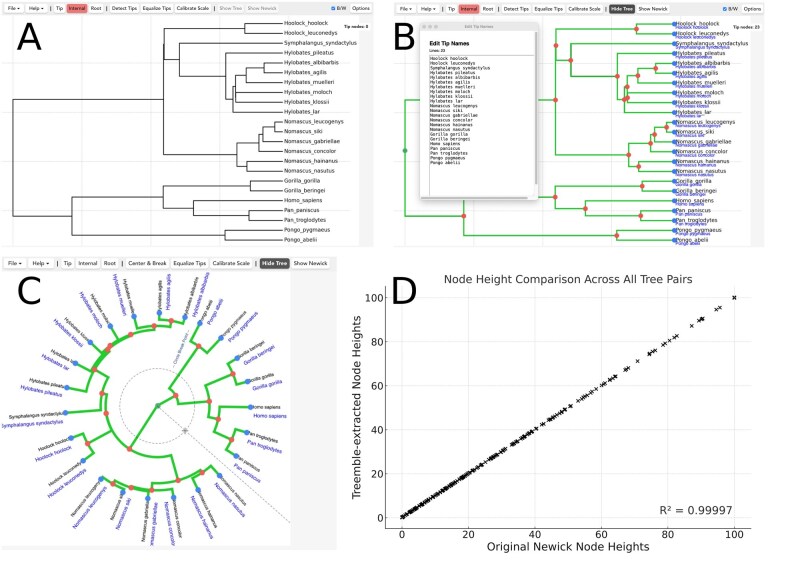
Treemble views and performance. (A) The Treemble interface with a primate tree figure loaded. (B) The Treemble interface with tip and internal nodes marked and green branch overlays depicting the connectivity of the algorithmically reconstructed tree shown. The tip name editor window is shown and name label overlays are shown in blue under each tip, allowing the user to check the spelling of the labels. (C) A circular tree is shown in the Treemble interface. The Show Tree option is on and green overlays overlap the tree image’s branches. (D) A comparison of node heights from original simulated trees and trees extracted using Treemble from figures of the originals. The $R^{2}$ is 0.99997, indicating an extremely accurate recovery of branch lengths across these trials.

Treemble provides a graphical interface for the user to click each node to mark it in the phylogeny display, which can be done in any order (Fig. 1). In addition, a deep learning-based node detection tool allows automatic marking of most nodes in typical published tree figures. Even when completed fully manually, we found the procedure to take approximately one second per node, which means that a phylogeny with 50 tips can be marked within two minutes.

After the user finishes marking the nodes, Treemble automatically determines the connectivity of the nodes (see Section 3 below) and displays an overlay depicting the tree it reconstructed (Fig. 1). This enables the user to verify that the phylogeny detected is correct. Treemble automatically highlights nodes that could not be fully connected, which can help to find missing nodes. Of course, a user may choose to only mark up a subset of nodes to produce a Newick tree of a subset of taxa.

Treemble measures branch lengths in pixels, which can be converted into biological units by calibrating the scale based on a visual scale bar. The tip names can be imported from a text file which can be generated by the user’s choice of any optical character recognition software. We provide Tip Name Extractor GPT, which is a customized AI Chatbot designed to read tip names from tree figures. It is based on OpenAI’s GPT framework; see openai.com/blog/introducing-gpts/. A user simply submits their tree image to the AI and receives a link to download a text file which can be dragged and dropped onto the Treemble window to load tip names. Names are displayed next to each tip so spelling can be checked and an editor allows adjustments to be made within Treemble (Fig. 1).

In addition to rectangular trees, Treemble has a specialized mode for circular trees, including circular scale calibration and radial branch lengths (Fig. 1). No previous software tool attempted to provide such a facility. Polytomies and freeform connectivities can be specified by the user by manually connecting nodes to the correct parent with a simple two-click interaction. Beyond capturing trees from the literature, Treemble can also be used in a blank canvas mode with drawing tools, so users can quickly sketch a tree by hand and create a Newick string according to their needs.

A cladogram mode allows any tree to be exported without branch lengths. An SVG image of any extracted tree can be exported directly. Full documentation with helpful visualizations is provided at Treemble.org, and a quickstart guide is available inside Treemble’s interface.

In order to test the accuracy of Treemble in recovering the branch lengths of trees from images, we generated ten simulated trees with 25 taxa each, using a lineage birth-death process as implemented in the DendroPy package (Sukumaran and Holder 2010). The resulting Newick strings were rendered as tree images using Biopython. Each image was loaded in Treemble, which was used to export a Treemble-extracted Newick string for the same tree. The resulting Newick strings all perfectly matched the original topology. The node heights of each internal node were compared between the original and Treemble-extracted versions (Fig. 1). The $R^{2}$ value was 0.99997, which indicates that the match is highly accurate. The total time taken to extract a Newick string from each simulated tree image was 2 minutes on average. For a comparison of branch lengths, the $R^{2}$ value was 0.99994. The mean absolute error was 0.1118 with a mean branch length of 19.6067 (0.57%). This is very likely to be well within the phylogenetic reconstruction uncertainty. The trials were performed using relatively low-resolution images (width = 1000 pixels), and high-resolution figures will lead to even better accuracy. We also simulated trees of up to 250 taxa in increments of 25 starting with 25, and the same test procedure was used. The $R^{2}$ values for branch lengths and node heights were all >0.9999 (see Fig. 1, available as supplementary data at *Bioinformatics* online). All tree images, data, and code used to perform these simulations and trials are available in the Dataset, available as supplementary data at *Bioinformatics* online. In summary, Treemble is a robust tool for recovering phylogenetic tree data from tree images.

## 3 Implementation

### 3.1 Software

Treemble is implemented as a native desktop application using the Tauri framework (v2.5.0). Tauri couples a small Rust-based backend with a system-native webview (WKWebView on macOS, WebView2 on Windows, and WebKitGTK on Linux), allowing the application logic and user interface to be written in TypeScript/React while retaining the size and performance characteristics of a compiled binary. We found the interface to be efficient enough to gather data from even very large trees, as we could easily complete a Newick string from a phylogeny of 1,382 tips (Larridon et al. 2021). Unlike Electron, where the runtime bundles an entire Chromium instance, resulting in binaries that are hundreds of megabytes, Tauri re-uses the host’s browser engine and links statically to Rust libraries. As a result, Treemble installers are small (approximately 20 megabytes).

### 3.2 Tree assembly algorithm

Treemble uses a novel algorithm to assemble an adjacency graph based on coordinates of nodes. The nodes are classified by the user as either tip or internal nodes. The algorithm iterates over the internal nodes from youngest to oldest (i.e. largest to smallest coordinate), connecting each one to the two younger parentless nodes that are nearest to it in the positive and negative directions, respectively. A high-level procedure follows:

1. Let *D* denote the set of all user-placed nodes (tip and internal). Initialize the set of free nodes F←D.
2. Collect the internal nodes
  (1) $$U=\{u\in D|{\text{type}}_{u}=\text{internal}\},$$

and sort them in strictly descending *x*.

1. For each internal node u∈U in that order:
2. Partition the current free set *F* into
  (2) $$F^{+}(u)=\{v\in F|x_{v}>x_{u},\;y_{v}>y_{u}\},$$
   
  (3) $$F^{-}(u)=\{v\in F|x_{v}>x_{u},\;y_{v}<y_{u}\}.$$
3. Choose
  (4) $$v^{+}=\arg\underset{v\in F^{+}(u)}{\min}(y_{v}-y_{u}),$$
   
  (5) $$v^{-}=\arg\underset{v\in F^{-}(u)}{\min}(y_{u}-y_{v}).$$
4. Attach edges $\left(u,v^{+}\right)$ and $\left(u,v^{-}\right)$ and remove $v^{+},v^{-}$ from *F*.
5. Continue until F=∅. A Newick string can be obtained by recursion on the adjacency graph.
6. Branch lengths, *L*, are obtained directly from the time axis:
  (6) $$L_{u\to v}=x_{v}-x_{u}.$$

### 3.3 Circular trees

For circular trees, the *X* and *Y* dimensions are simply transformed into polar coordinates and the same algorithm applies, following the designation of a center point by the user.

Let the user-chosen center point and break angle be $\left(c_{x},c_{y}\right)$ and $\theta_{\text{break}}$ respectively. Convert each screen coordinate (x,y) to polar coordinates by


(7) $$r=\sqrt{{\left(x-c_{x}\right)}^{2}+{\left(y-c_{y}\right)}^{2}},$$

(8) $$\theta =\theta_{\text{break}}-\mathrm{atan}2(y-c_{y},x-c_{x}).$$

Then the exact same algorithm from Section 3.2 applies, with


(9) $$F^{\text{cw}}(u)=\{v\in F|r_{v}>r_{u},\;0<((\theta_{v}-\theta_{u})\mathrm{mod}2\pi )\},$$

(10) $$F^{\text{ccw}}(u)=\{v\in F|r_{v}>r_{u},\;0<((\theta_{u}-\theta_{v})\mathrm{mod}2\pi )\}.$$

The nearest clockwise (CW) and counter-clockwise (CCW) descendants are then chosen by minimizing the positive modular angular difference. $\theta_{\text{break}}$ is used as the starting point for adding tip-name labels.

### 3.4 Node detection model

A convolutional neural network was trained to assist users by automatically detecting nodes. The accuracy is insufficient for complete automation, but users have reported substantial time savings over fully manual node marking even allowing for corrections that must be made. The model predicts a dense per-pixel heatmap of nodes from rasterized tree crops. During inference the heatmap is converted to candidate coordinates by non-maximum suppression and thresholding. The model was trained on a curated set of over 500 published rectangular tree figures annotated manually using Treemble by trained human curators. See Methods, available as supplementary data at *Bioinformatics* online, for more details on model architecture, training, and inference.

## 4 Discussion

Treemble provides a unique solution to the problem of recovering machine-readable representations of phylogenetic trees from images. It may also be useful beyond phylogenetics for transcribing trees representing populations, languages, cell lineages, and other such cases across disciplines. A typical user should be able to generate a Newick string, including tip names, with Treemble in about 1 minute for every 10 taxa in a standard tree, based on our experience using Treemble with hundreds of timetrees. In these efforts, verifying tip names takes longer than acquiring the branching pattern. Treemble is limited by the readability of tree figures for humans. Very dense tree figures whose branches fully overlap cannot be captured by Treemble, but it can often be used to acquire a backbone tree from such figures.

Treemble offers many quality-of-life features for users, including image zoom for detailed node placement, undo and redo support, keyboard shortcuts, autosave and session recovery, tools for performing a diff analysis to compare two sets of tip names, automatic equalization of tip positions for ultrametric trees, grayscale mode to improve node visibility, a range of visual style options, and system dark mode support. The ability to save a CSV file of node locations, which can include tip names, allows a Treemble session to be reopened and modified. Treemble’s simplicity and versatility will enable analyses of published phylogenies that were previously prohibitively difficult, paving the way for new advances in phylogenetics.

## Supplementary Material

btag197_Supplementary_Data

## Data Availability

Installers for Treemble for macOS and Windows are freely available for download at treemble.org, where full illustrated documentation can also be found. Source code for Treemble is available at github.com/John-Allard/Treemble. The trained node detection model is freely available and documented at huggingface.co/John-Allard/treemble-1.
