# Supplementary material for: Treemble: a graphical tool to generate Newick strings from phylogenetic tree images
Source: Bioinformatics. 2026 Apr 22;42(5):btag197. doi: 10.1093/bioinformatics/btag197 (PMC13171175; doi:10.1093/bioinformatics/btag197)
Supplement: btag197_Supplementary_Data [file btag197_supplementary_data.zip › treemble_supplementary_information.pdf]

Treemble: A Graphical Tool to Generate Newick Strings from Phylogenetic Tree Images

Supplementary information

John B. Allard[1,2]* and Sudhir Kumar[1,2]

**Corresponding author*

john.allard@temple.edu

Affiliations

[1] Institute for Genomics and Evolutionary Medicine, Temple University, Philadelphia, PA 19122, USA

[2] Department of Biology, Temple University, Philadelphia, PA 19122, USA

Supplementary methods

Automated Internal Node Detection in Treemble

S1. Task overview
Treemble's internal node detector is a heatmap-based keypoint model that takes a cropped tree figure region as input and outputs a dense per-pixel probability map (heatmap). Local maxima of this heatmap correspond to candidate internal node locations, which are mapped back to the original tree image and presented to the user for review and editing.

S2. Data source and annotation
Training data consisted of phylogenetic tree figures collected from the scientific literature. All trees were rectangular ultrametric time-calibrated species phylogenies of clades across the entire tree of life. All figures were manually annotated with node coordinates (tip, internal, root) by paid research assistants in our lab, with multiple rounds of human review as part of the core annotation workflow. Due to publisher copyright restrictions, the full image dataset cannot be redistributed. The internal node detector was trained only on the cropped portions of these figures that contained the tree and its annotations.

S3. Dataset preparation and filtering
Each sample includes:
(i) a raster image of a tree, and
(ii) a CSV of node annotations with coordinate columns x, y and a type label.
Figures were cropped to the tight bounding box of annotated nodes with a 15-pixel margin, and node coordinates were shifted accordingly. To reduce noise from very dense tip clusters that often overlap visually, figures with extremely dense tip labeling that violated a minimum vertical tip spacing criterion were excluded to avoid cases where tip labels visually obscure internal nodes. Additionally, only trees with a single root and a leftmost root position (consistent with a rooted left-to-right layout) were retained for training. After filtering, the dataset comprised 541 figures with a total of 57,983 annotated nodes, of which 28,721 were internal or root nodes.

S4. Splitting
The filtered dataset was split randomly into training (90%) and validation (10%) sets using a fixed seed (1337) for reproducibility. No external held-out test set was defined for this run (see S10).

S5. Preprocessing
All crops were converted to single-channel grayscale and normalized to [0,1]. Images were resized such that the longer side did not exceed 2000 pixels while preserving aspect ratio, then zero-padded to dimensions divisible by 32 to match the network's downsampling and upsampling structure.

S6. Target generation

Ground-truth node annotations in the resized and padded image space were converted into a single internal-node heatmap channel by placing 2D Gaussian kernels ($\sigma = 1.5$ pixels) at each internal and root coordinate.

S7. Model

The model consists of a ResNet-34 (He et al., 2016) encoder with ImageNet-pretrained (Deng et al., 2009) weights adapted to one grayscale input channel, followed by a U-Net (Ronneberger et al., 2015) style decoder to produce a full-resolution heatmap. The first encoder stage was frozen during training. Details of the ONNX-exported model and its associated inference configuration are documented in the public model repository.

S8. Training

The network was trained using the AdamW optimizer (Loshchilov and Hutter, 2019) with an initial learning rate of 1×10^{-4}, cosine annealing to 1×10^{-5}, and a batch size of 1. Heatmap logits were trained with weighted binary cross-entropy loss emphasizing positive Gaussian regions, with a positive weight of 25.0. Photometric augmentations (random brightness and contrast jitter) were applied during training. The best checkpoint was selected based on minimum validation loss with early stopping (patience 10 epochs), and training completed in under a day on an NVIDIA 4080 Super GPU. Typical inference on a single crop runs in approximately one second on a CPU.

S9. Inference decoding

At inference time, the ONNX-exported model (together with a `model.config.json` encoding preprocessing and decoding parameters) is used to produce a heatmap, which is passed through a sigmoid and subjected to non-maximum suppression (7×7 window). A probability threshold of 0.3 and peak limiting (max 2000 peaks per channel, 5000 per image) yield candidate internal node coordinates for display and manual editing. The trained ONNX model and configuration are available under the MIT license at the Hugging Face model hub.

S10. Evaluation

The model is designed as an assistive component within an interactive graphical workflow rather than as a fully autonomous predictor. Users visually inspect model-proposed node locations and manually correct or add nodes as needed before exporting a Newick representation. Because correctness of the final tree structure is verified by the user during this process, we used a 90/10 training and validation split primarily for model selection and convergence monitoring. Validation metrics were computed during training to guide hyperparameter selection and checkpoint choice. In practice, the deployed model has performed robustly on newly encountered tree figures not included in the training data, as assessed through routine use within the Time Tree of Life curation workflow.

Supplementary Figures

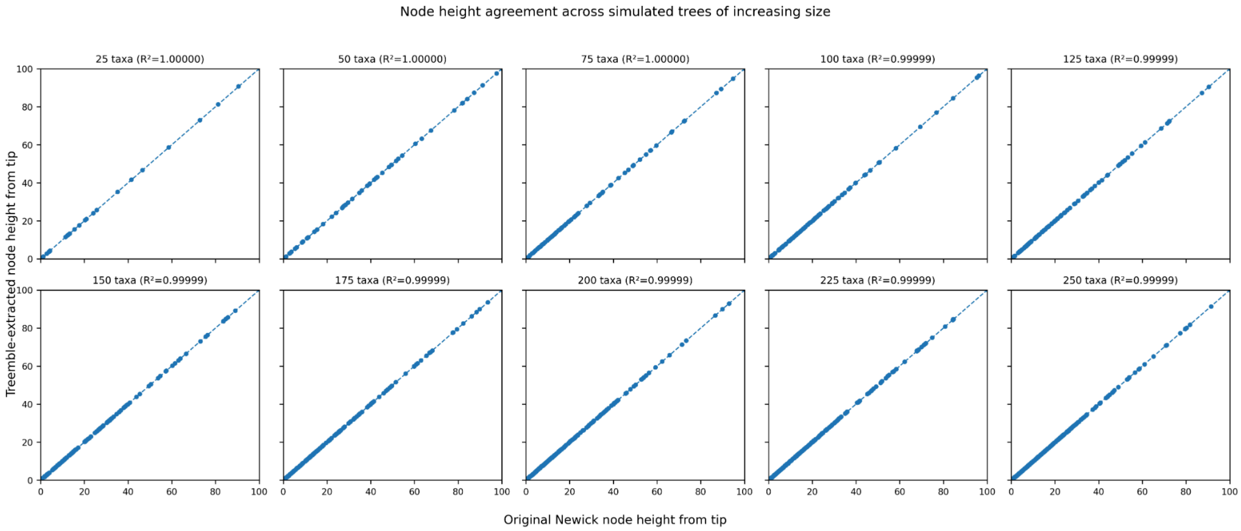

Supplementary Figure 1. Node height agreement between original and Treemble-extracted Newick strings for simulated ultrametric trees with 25 to 250 taxa (increments of 25). Each panel shows internal node heights from tip in the original Newick versus the corresponding heights in the Treemble-extracted Newick. The dashed line indicates equality (y = x).

Supplementary dataset

The supplementary dataset (treemble_benchmarking_data.zip) contains the simulated trees, rendered images, Treemble node location CSVs, extracted Newick strings, and scripts used to evaluate Treemble on simulated ultrametric trees, including (i) ten replicate 25-taxon simulations and (ii) single-tree simulations for taxa counts from 25 to 250 in increments of 25.

Supplementary references

He,K., Zhang,X., Ren,S. and Sun,J. (2016) Deep residual learning for image recognition. Proc. IEEE Conf. Comput. Vis. Pattern Recognit. (CVPR), 770–778.

Ronneberger,O., Fischer,P. and Brox,T. (2015) U-Net: Convolutional networks for biomedical image segmentation. In: MICCAI, 234–241.

Deng,J. et al. (2009) ImageNet: A large-scale hierarchical image database. Proc. IEEE Conf. Comput. Vis. Pattern Recognit. (CVPR), 248–255.

Loshchilov,I. and Hutter,F. (2019) Decoupled weight decay regularization. In: Int. Conf. Learn. Represent. (ICLR).
